# Galangin Regulates Oxidative Stress Levels in Porcine Embryos Through Interaction with the Neh1 Domain of Nrf2

**DOI:** 10.3390/antiox14070822

**Published:** 2025-07-04

**Authors:** Zhi-Chao Chi, Shu-Ming Shi, Li-Ying Liu, Lin-Yi Qu, Jing-Hang Li, Guan-Lin Jia, Yu-Yan He, Lin-Xuan Li, Yong-Xun Jin, Ming-Jun Zhang, Xian-Feng Yu

**Affiliations:** College of Animal Sciences, Jilin University, Changchun 130062, China; chizc23@mails.jlu.edu.cn (Z.-C.C.); shism20@mails.jlu.edu.cn (S.-M.S.); liuliying22@mails.jlu.edu.cn (L.-Y.L.); quly23@mails.jlu.edu.cn (L.-Y.Q.); jinghang23@mails.jlu.edu.cn (J.-H.L.); jiagl23@mails.jlu.edu.cn (G.-L.J.); heyy23@mails.jlu.edu.cn (Y.-Y.H.); lxli9922@mails.jlu.edu.cn (L.-X.L.); jyx0429@jlu.edu.cn (Y.-X.J.); mjzhang@jlu.edu.cn (M.-J.Z.)

**Keywords:** galangin, IVC, preimplantation embryo, Nrf2, oxidative stress, mitochondria

## Abstract

Oxidative stress poses a challenge to in vitro embryo culture. As a flavonoid, galangin (GAL) has been shown to have antioxidant effects, but the effect and antioxidant capacity of GAL in the in vitro development of porcine parthenogenetic embryos are still unknown. In this study, we demonstrated that 1 µM GAL significantly increased the blastocyst rate, decreased the accumulation of intracellular reactive oxygen species (ROS), increased the glutathione (GSH) level, and enhanced mitochondrial function in early porcine embryos. Nuclear factor erythroid-2-related factor 2 (*Nrf2*) was identified as the target gene of GAL via network pharmacology, and the transcript levels of related antioxidant enzymes (HO-1, NQO1, SOD2, and CAT) were found to be increased. Since Nrf2 has seven domains, we constructed Nrf2 mutants lacking different domains in vitro. We found that GAL specifically binds to the Neh1 domain of Nrf2. Subsequent embryonic experiments demonstrated that the antioxidant effect of GAL was abolished after Nrf2 deletion. These results suggest that GAL can directly bind to Nrf2 to regulate the level of oxidative stress and improve mitochondrial function in embryos.

## 1. Introduction

In vitro culture (IVC) is an indispensable step in the in vitro production (IVP) of live embryos. Embryos obtained through IVC can be used for a variety of purposes, such as producing offspring [1] or for research [2,3] on embryonic developmental mechanisms. However, mimicking the IVP environment remains difficult [4], causing the developmental potential and quality of in vitro-produced embryos to be lower than those of in vivo embryos [5]. Therefore, optimizing the IVC system for embryos is very important.

The accumulation of ROS during IVC is one of the main factors affecting the efficiency of embryo production [6]. High levels of ROS cause DNA damage [7], decreased mitochondrial function [8], and structural changes in proteins [9] in embryos, thus affecting the quality of the embryo. Compared with other species, pigs have high endogenous lipid contents [10], and ROS are generated during the oxidation of endogenous fatty acids to provide energy [11]. Therefore, in vitro porcine embryos are more sensitive than other embryos to oxidative stress. Currently, the application of antioxidants, such as resveratrol [12], melatonin [13], and vitamin C [14], is the main approach for mitigating ROS-mediated damage to porcine embryos during IVC. However, the effects of other antioxidants still need to be explored to establish a better-quality culture system.

Galangin (GAL) is a natural flavonoid that is abundant in galangal and propolis. GAL has been shown to play a role in a variety of biological processes, including the resolution of inflammation [15], the antioxidant system [16], gastrointestinal diseases [17], and cancer [18]. Although the antioxidant effects of GAL have been widely studied and reported, the effects of GAL on early embryo development in pigs are still unknown.

The nuclear factor erythroid-2-related factor 2 (Nrf2)/Kelch-like ECH-associated protein 1 (Keap1) signaling pathway regulates intracellular antioxidative stress and metabolism and plays an essential role in maintaining cellular homeostasis and resisting harmful external substances [19]. Nrf2 is a transcription factor normally found in the cytoplasm [20]. In the absence of oxidative stress, Nrf2 binds to Keap1 in the cytoplasm, promoting the ubiquitination and subsequent proteasomal degradation of Nrf2. When cells are stimulated by oxidative stress, cysteine residues of Keap1 are modified, leading to a conformational change in Keap1 and the release of Nrf2 [21,22]. Nrf2 subsequently translocates to the nucleus and binds to antioxidant response elements (AREs), which activate the expression of downstream antioxidant enzymes, such as superoxide dismutase (SOD2), catalase (CAT), NAD(P)H quinone oxidizing reductase 1 (NQO1), and heme oxygenase 1 (HO-1) [23,24,25,26,27,28,29,30]. The Nrf2 protein contains seven conserved structural domains, named Neh (Nrf2-Echinoid homolog homology) domains (Neh1-Neh7), each with a unique function [31]. The Neh1 domain contains the NLS sequence, which is required for the nuclear localization of Nrf2 [32]. The Neh2 domain contains the “DLG” and “ETGE” motifs, which are binding sites for Nrf2 to Keap1 [33]. Neh3-5 contain trans-activating structural domains for gene transcription that bind to transcriptional co-activators [34,35], and Neh7 has been shown to inhibit the Nrf2-ARE signaling pathway [36].

The aim of this study was to investigate the antioxidant effect of GAL on porcine parthenogenetic embryos and to investigate the mechanism underlying this antioxidant effect. Network pharmacology analysis was used to identify the target genes through which GAL exerts its antioxidant effects. Experiments involving the in vitro construction of mutants lacking structural domains subsequently revealed the specific structural domains through which GAL binds to its target genes, and embryonic experiments further demonstrated that GAL and its target genes bind to each other to exert their effects. Using these methods, we explored the potential mechanism underlying the antioxidant effects of GAL.

## 2. Materials and Methods

All reagents were purchased from Sigma Aldrich (St. Louis, MO, USA) unless otherwise indicated.

### 2.1. Oocyte Collection and In Vitro Maturation (IVM)

Porcine ovaries were obtained from a local slaughterhouse, preserved in 0.9% saline containing penicillin–streptomycin at 37 °C, and delivered to the laboratory within 2 h. Follicular fluid was obtained by aspirating follicles 3–6 mm in diameter using a sterile syringe. The resulting follicular fluid was placed in a 10 mL centrifuge tube and allowed to settle for 8 min. The supernatant was aspirated with a pasteurized tube, and the precipitate was washed with HEPES 3 times. The cumulus–oocyte complexes (COCs) encapsulated by 2–3 layers of cumulus cells were removed.

IVM was performed using four-well plates, and 500 µL of culture medium and 50 COCs were added to each well and covered with mineral oil. Medium 199 containing 10% porcine follicular fluid, 0.01 mg/mL sodium pyruvate, 0.01 µg/mL EGF, PMSG (10 IU/mL), and HCG (10 IU/mL) was used as the basal medium. The oocytes were incubated at 38.5 °C in a 5% CO_2_ incubator for 44 h. After 44 h, cumulus cells were removed with 0.2% hyaluronidase, and then oocytes that had extruded the first polar body were collected for further experiments.

### 2.2. Parthenogenetic Activation and IVC

Selected oocytes were utilized for the production of parthenogenetic embryos using electroactivation. Parthenogenetic activation was conducted by applying two direct current pulses of 120 V/mm for 60 μs using an electrofusion instrument (ECFG21, NEPA GENE, Kashiwa, Chiba, Japan). After electroactivation, they were transferred to 7.5 µg/mL cytochalasin B for 3 h to inhibit the expulsion of dipolar bodies. The activated oocytes were subsequently transferred to IVC medium (PZM-5) and cultured in an incubator at 38.5 °C in 5% CO_2_. The cleavage rate was determined after 24 h, and the blastocyst rate was determined on day 6.

### 2.3. Treatment with GAL

GAL was dissolved in dimethyl sulfoxide (DMSO) and further diluted with IVC medium so that the concentration in the culture solution was 1, 2, or 4 µM. The dilution of DMSO in the experimental group was 1/1000 throughout the experiment. The dilution of DMSO in the control group was also 1/1000.

### 2.4. Embryo Microinjection

After parthenogenetic activation of mature oocytes, *si-Nrf2* was injected into one-cell-stage embryos using an electron microsyringe pump (Eppendorf, FemtoJet 4i, Hamburg, Germany), and controls were injected with ddH_2_O. The *si-Nrf2* sequences were as follows: sense (5′-3′)-CGACAGAAAUUGACAACUAUC; antisense (5′-3′)-UAGUUGUCAAUUUCUGUCGGU. After injection, half of the embryos were placed in IVC medium containing GAL, and the other half were left untreated. The embryos were cultured until day 6 in an incubator containing 5% CO_2_.

### 2.5. Measurement of Intracellular ROS and Glutathione GSH Levels

Four-cell and day 6 blastocysts were collected to measure ROS and GSH levels, respectively. The embryos were washed 3 times with PBS-PVA (0.1% PVA) and then incubated with 10 mM 2’,7’-dichlorodihydrofluorescein diacetate (Invitrogen, C400, Carlsbad, CA, USA) or 10 mM 4-chloromethyl-6,8-difluoro-7-hydroxycoumarin (Invitrogen, C12881, Carlsbad, CA, USA) for 30 min. Photographs were taken using fluorescence microscopy (green fluorescence, 490 nm; blue fluorescence, 370 nm). Fluorescence intensity analysis was performed using ImageJ (v1.54e, National Institutes of Health, Bethesda, MD, USA).

### 2.6. Measurement of the Mitochondrial Membrane Potential

Embryos at the four-cell period were harvested, washed 3 times with PBS-PVA, and then incubated with 2 mM JC-1 dye (Beyotime, C2006, Shanghai, China) for 30 min, after which the cells were washed again 3 times with PBS-PVA. Red and green fluorescence were imaged using a fluorescence microscope equipped with a camera, and the red fluorescence/green fluorescence intensity ratio was analyzed using ImageJ (v1.54e, National Institutes of Health, Bethesda, MD, USA).

### 2.7. Cell Proliferation and Apoptosis Assays

Blastocysts were collected on day 6 and washed 3 times using PBS-PVA, and the proliferation of blastocysts was examined using EDU dye according to the manufacturer’s instructions (Beyotime, C0071L, Shanghai, China). After staining, the blastocysts were incubated for 10 min at room temperature in a solution containing Hoechst 33342 (Beyotime, C1027, Shanghai, China). A fluorescence microscope was used to take pictures, and ImageJ (v1.54e, National Institutes of Health, Bethesda, MD, USA) was used to assess cell proliferation. Blastocysts were collected on day 7, washed 3 times with PBS-PVA, and then incubated with 50 µL of TUNEL (Beyotime, C1086, Shanghai, China) reaction mixture for 1 h at 37 °C in the dark. After staining was complete, the samples were incubated for 10 min at room temperature in a solution containing Hoechst 33342. Photographs were taken using a fluorescence microscope, and ImageJ (v1.54e, National Institutes of Health, Bethesda, MD, USA) was used for statistical analysis of cell apoptosis.

### 2.8. Counting the Number of Blastocyst Cells

To count the number of blastocyst cells, blastocysts were collected on day 6 and fixed in 4% paraformaldehyde for 30 min. The blastocysts were washed 3 times in PBS-PVA and then placed in PBS-PVA containing 0.1% Triton X-100 for 30 min. After 3 washes with PBS-PVA, the blastocysts were incubated in a solution containing 5 ng/mL Hoechst 33342 for 10 min at room temperature. The blastocysts were then placed on slides and sealed with antifluorescence quenching reagents. Photographs were taken using a fluorescence microscope equipped with a camera, and ImageJ (v1.54e, National Institutes of Health, Bethesda, MD, USA) was used to count the number of cells.

### 2.9. Quantitative Real-Time PCR (qRT–PCR)

Day 7 blastocysts were collected, and RNA was extracted from 100 blastocysts per group. RNA was extracted using an RNA Mini Kit (Qiagen, 80284, Hilden, Germany). qRT–PCR was performed via a three-step process using the reaction system described in the instructions of the Monad’s MonAmp^TM^ ChemoHS qPCR Mix Kit. All components were mixed thoroughly, and qRT–PCR was performed using a three-step method. The PCR primer sets employed for gene amplification are detailed in Table 1.

### 2.10. Identification of GAL Target Genes and Antioxidant-Related Targets

The Swiss Target Prediction (http://www.swisstargetprediction.ch/) (accessed on 18 August 2024), PharmMapper (http://lilab-ecust.cn/pharmmapper) (accessed on 18 August 2024), and SuperPred (http://prediction.charite.de/subpages/target_prediction.php) (accessed on 18 August 2024) databases were used to identify gene targets of GAL. The Gene Ontology (GO) database was subsequently searched for antioxidant-related genes with the keyword “GO:0006979” (accessed on 18 August 2024). Finally, the GAL target genes and antioxidant genes were intersected to identify the intersecting targets. A Venn diagram was generated using the “VennDiagram” package (v1.7.3) in R (v4.4.0) to visualize the intersecting genes.

### 2.11. GO and Kyoto Encyclopedia of Genes and Genomes (KEGG) Enrichment Analyses

GO and KEGG enrichment analyses were performed via R packages such as Cluster Profiler (v3.14.3), ggplot2 (v3.5.1), openxlsx (v4.2.6), and tidyverse (v2.0.0). Visualization was achieved using bar charts.

### 2.12. Construction of Protein–Protein Interaction (PPI) Networks

The intersecting targets were imported into the STRING database (http://string-db.org/) (accessed on 19 August 2024) to construct a PPI network to evaluate the interactions between targets. The PPI network was subsequently analyzed using Cytoscape 3.9.1 (National Resource for Network Biology, La Jolla, CA, USA) to identify the core targets using the median degree, closeness, and betweenness values. The obtained core targets were visualized using Cytoscape 3.9.1 (National Resource for Network Biology, La Jolla, CA, USA).

### 2.13. Molecular Docking Analysis

The structure of the Nrf2 protein was downloaded from the PDB database (accessed on 4 January 2025), and the ligand and protein fractions were isolated. The structures of small-molecule compounds were obtained from the PubChem database (accessed on 4 January 2025). Docking was performed using AutoDock Vina (v1.2.5). The binding site with the lowest capacity was considered the most potent binding site. Finally, the docking conformations were visualized using PyMOL (v3.0.0).

### 2.14. Cellular Thermal Shift Assay (CETSA)

After the protein was extracted from the collected embryos or bacterial fluids, the protein samples were incubated with 1 mM GAL or DMSO as a control for 1 h at room temperature, heated at different temperatures (40–80 °C) for 3 min, and cooled at 4 °C for 4 min. Next, the samples were centrifuged at 12,000× *g* for 30 min, the supernatant was aspirated, and target protein levels were measured via Western blotting.

### 2.15. Drug Affinity Responsive Target Stability (DARTS) Assay

After protein was extracted from blastocysts, the samples were incubated with 1 mM GAL or DMSO as a control for 1 h at room temperature. Subsequently, streptavidin was added at a streptavidin/total protein ratio of 1:500, 1:1000, or 1:2000 (*w*/*w*), and the samples were incubated at 37 °C for 30 min. The reaction was terminated by adding a loading buffer and heating at 95 °C for 10 min, followed by measurement of target protein levels via Western blotting.

### 2.16. Expression of Recombinant Nrf2

DNA sequences of full-length Nrf2 and 6 Nrf2 mutants of different lengths were ligated to the pET-28a(+) plasmid vector (NotI/XhoI) with a His tag. BL21 (DE3) receptor cells were transformed with the recombinant plasmid. The receptor cells were plated in LB liquid medium and incubated at 37 °C and 180 rpm until the OD 600 reached 0.6. The cells were subsequently stimulated with isopropyl β-D-1-thiogalactopyranoside (IPTG) at a final concentration of 0.5 mM and incubated for 15 h at 37 °C. After the bacterial fluid was collected, protein was extracted using a bacterial protein extraction kit (Sangon Biotech, C600596, Shanghai, China), and target protein levels were measured via Western blotting.

### 2.17. Western Blotting

One hundred blastocysts were collected, and protein was extracted with RIPA buffer (Beyotime, P0013K, Shanghai, China). For the extraction of nuclear proteins, a commercial kit was used to separate nuclear proteins from cytoplasmic proteins according to the instructions (Epizyme Biotech, PC204, Shanghai, China). Next, the protein samples were separated on a 12% polyacrylamide gel containing 0.1% SDS and transferred to polyvinylidene difluoride membranes (Millipore, ISEQ00010, Billerica, MA, USA). After the membranes were blocked with protein-free rapid sealing buffer (1×) for 30 min, they were incubated with a rabbit anti-Nrf2 antibody (1:1000, Proteintech, 16396-1-AP, Rosemont, IL, USA), rabbit anti-Keap1 antibody (1:5000, Proteintech, 50,599-2-Ig, Rosemont, IL, USA), anti-HA tag antibody (1:2000, Proteintech, 51064-2-AP, Rosemont, IL, USA), anti-Histone H3 antibody (1:2000, Proteintech, 68345-1-Ig, Rosemont, IL, USA), or beta-actin rabbit antibody (1:5000, Cell Signaling Technology, 4967, Danvers, MA, USA) at 4 °C overnight. The membranes were then washed 3 times in TBST for 10 min each. The membranes were incubated with horseradish peroxidase-coupled goat anti-rabbit IgG (1:5000, Bioworld Technology, BS13278, St. Louis Park, MN, USA) for 1 h at room temperature. Finally, the membranes were washed 3 times in TBST. The blots were visualized using a Tanon 5200 image analyzer (Tanon, Shanghai, China) and analyzed via ImageJ (v1.54e, National Institutes of Health, Bethesda, MD, USA) software.

### 2.18. Immunofluorescence

Blastocysts were fixed in 4% paraformaldehyde for 30 min, washed 3 times in PBS-PVA, placed in PBS-PVA containing 0.1% Triton X-100 for 30 min, and then incubated in 5% BSA for 1 h. The blastocysts were incubated with primary antibody (rabbit anti-Nrf2 antibody (1:200, Proteintech, 16396-1-AP, Rosemont, IL, USA), rabbit anti-Keap1 antibody (1:200, Proteintech, 50,599-2-Ig, Rosemont, IL, USA)) overnight at 4 °C and then with secondary antibody (Abbkine, A23220, Wuhan, China) for 1 h in the dark. The blastocysts were then incubated in 5% BSA for 1 h. The blastocysts were incubated with primary antibody overnight at 4 °C and then with secondary antibody in the dark for 1 h. After staining, the blastocysts were incubated in a solution containing Hoechst 33342 for 10 min at room temperature. Photographs were taken using a fluorescence microscope and statistically analyzed with ImageJ (v1.54e, National Institutes of Health, Bethesda, MD, USA).

### 2.19. Statistical Analysis

In this study, all the experiments were repeated 3 times. The data were statistically analyzed using GraphPad Prism 9 (GraphPad Software, San Diego, CA, USA), and comparisons between groups were made using independent t tests or one-way analysis of variance (ANOVA). Post hoc analyses were conducted using Tukey’s multiple comparisons test to identify noteworthy differences between groups. *p* < 0.05 was considered to indicate statistical significance. The results are presented as the mean ± SEM.

## 3. Results

### 3.1. GAL Improves the Development of Porcine Parthenogenetic Embryos

**Figure 1:**
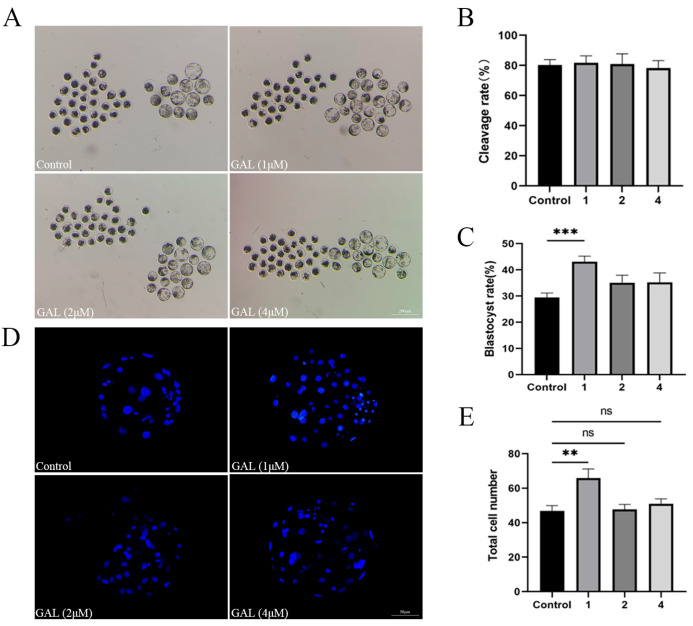
Effects of different concentrations of GAL (0, 1, 2, 4 µM) on porcine parthenogenetic embryo development. (**A**) Representative images of porcine parthenogenetic embryo development on day 6 in the control and GAL-treated groups. Scale bar = 200 µm. (**B**) Cleavage rate of porcine parthenogenetic embryos after 24 h (control: *n* = 262; 1: *n* = 265; 2: *n* = 270; 4: *n* = 266). (**C**) Blastocyst formation rate of porcine parthenogenetic embryos on day 6 (control: *n* = 262; 1: *n* = 265; 2: *n* = 270; 4: *n* = 266). (**D**) Representative fluorescence images of Hoechst-stained blastocysts from the control and GAL-treated groups on day 6. Scale bar = 50 µm. (**E**) Total cell number in blastocysts on day 6 (control: *n* = 71; 1: *n* = 70; 2: *n* = 75; 4: *n* = 71). Significant differences are indicated by ** (*p* < 0.01) and *** (*p* < 0.001). No significant difference (*p* > 0.05) is indicated by “ns”.

**Figure 2:**
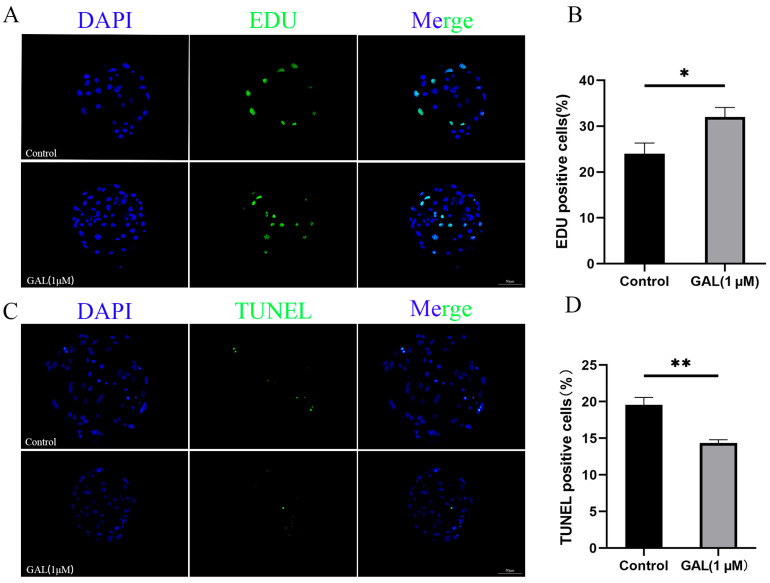
Effects of 1 µM GAL supplementation on cell proliferation and apoptosis in porcine parthenogenetic embryos at the blastocyst stage. (**A**) Representative images of EDU-positive cells in day 6 blastocysts. Scale bar = 50 µm. (**B**) Percentage of EDU-positive cells in day 6 blastocysts (control: *n* = 53; GAL: *n* = 55). (**C**) Representative images of TUNEL-positive cells in day 7 blastocysts. Scale bar = 50 µm. (**D**) Apoptosis rate of day 7 blastocysts (control: *n* = 62; GAL: *n* = 60). Significant differences are indicated by * (*p* < 0.05) and ** (*p* < 0.01).

To investigate the effects of GAL on early porcine embryos, different concentrations of GAL (0, 1, 2, 4 µM; more concentrations showed in Figure S2) were applied to porcine parthenogenetic embryos, and the effects of GAL on embryonic development were investigated. Compared with that in the control group, the blastocyst rate of porcine parthenogenetic embryos in the 1 µM GAL-treated group was significantly increased on day 6 (Figure 1A,C; 29.46% ± 1.71%, 43.09% ± 2.16%, 35.05% ± 2.94%, 35.24% ± 3.60%, *p* < 0.001), but there was no difference in the cleavage rate at 24 h (Figure 1B; *p* > 0.05; more concentrations showed in Figure S1). Day 6 embryos were harvested and stained to determine the number of blastocyst cells, and 1 µM GAL was found to increase the number of blastocyst cells (Figure 1D,E; *p* < 0.01). Further experiments revealed that, compared with the control treatment, 1 µM GAL increased proliferation (Figure 2A,B; *p* < 0.05) and decreased apoptosis (Figure 2C,D; *p* < 0.01) in porcine parthenogenetic embryos. On the basis of these experimental results, 1 µM was selected as the optimal concentration of GAL.

### 3.2. GAL Optimizes Mitochondrial Function

**Figure 3:**
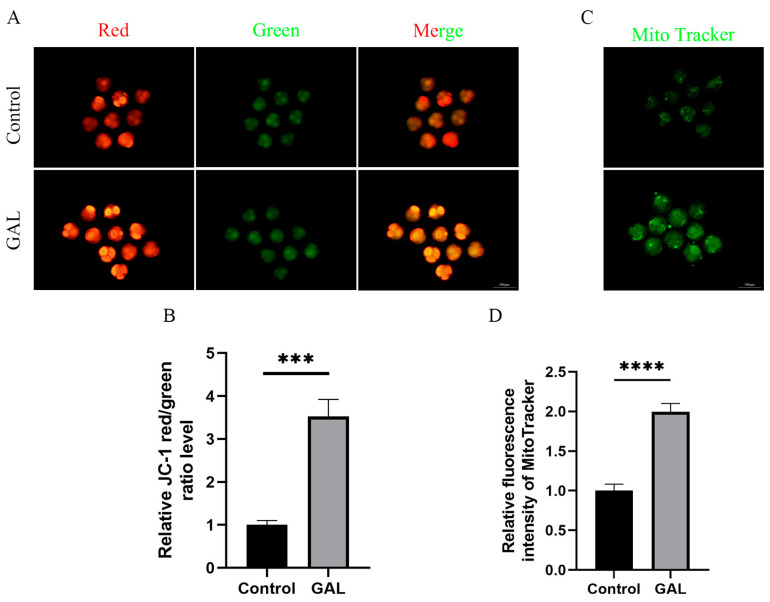
Effects of GAL supplementation on mitochondrial function in porcine parthenogenetic embryos. (**A**) Fluorescence images of JC-1 at the four-cell stage in the control and GAL-treated groups. Scale bar = 100 µm. (**B**) Relative fluorescence intensity of intracellular JC-1 at the four-cell stage (control: *n* = 74; GAL: *n* = 79). (**C**) Fluorescence images of MitoTracker staining at the four-cell stage in the control and GAL-treated groups. Scale bar = 100 µm. (**D**) Relative fluorescence intensity of MitoTracker at the four-cell stage (control: *n* = 96; GAL: *n* = 92). Significant differences are indicated by *** (*p* < 0.001) and **** (*p* < 0.0001).

Mitochondria provide energy for early embryonic development in pigs and are indispensable organelles for maintaining normal embryonic development. The mitochondrial content and membrane potential can be used as indicators to assess mitochondrial function. Therefore, we analyzed the mitochondrial content and membrane potential of porcine parthenogenetic embryos at the four-cell stage. The results revealed that the JC-1 red/green fluorescence intensity ratio increased significantly after GAL treatment (Figure 3A,B; *p* < 0.001), and the mitochondrial content also increased significantly (Figure 3C,D; *p* < 0.0001). These results indicate that GAL treatment can improve the mitochondrial function of porcine parthenogenetic embryos.

### 3.3. GAL Increases the Antioxidant Capacity of Early Porcine Embryos

**Figure 4:**
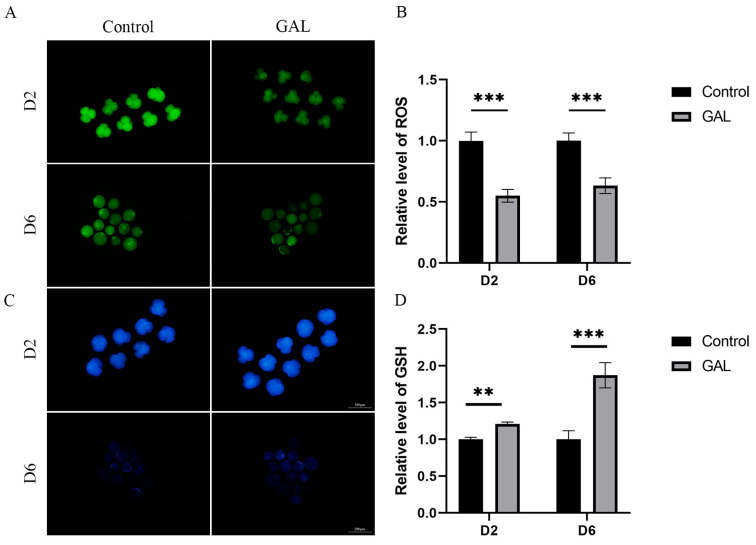
Effect of GAL supplementation on the antioxidant capacity of porcine parthenogenetic embryos. (**A**) Representative images of intracellular ROS in day 2 and day 6 embryos. D2: scale bar = 100 µm. D6: scale bar = 200 µm. (**B**) Relative intracellular ROS levels in day 2 and day 6 embryos (D2: control: *n* = 86; GAL: *n* = 83; D6: control: *n* = 42; GAL: *n* = 46). (**C**) Representative images of intracellular GSH in embryonic cells on days 2 and 6. D2: scale bar = 100 µm. D6: scale bar = 200 µm. (**D**) Relative intracellular GSH levels in embryonic cells on days 2 and 6 (D2: control: *n* = 77; GAL: *n* = 81; D6: control: *n* = 51; GAL: *n* = 56). Significant differences are indicated by ** (*p* < 0.01) and *** (*p* < 0.001).

To assess the ability of GAL to combat oxidative stress during the early development of porcine embryos, we examined ROS and GSH levels in four-cell (day 2) and day 6 blastocysts. The results revealed that ROS levels in four-cell and day 6 blastocysts were lower in the GAL-treated group than in the control group (Figure 4A,B; *p* < 0.001), whereas GSH levels were greater in the GAL group than in the control group at both stages of embryogenesis (Figure 4C,D; D2, *p* < 0.01; D6, *p* < 0.001). These results suggest that GAL increases the antioxidant capacity of porcine parthenogenetic embryos during development, resulting in the production of higher-quality embryos.

### 3.4. Predicting Potential Targets Through Which GAL Exerts Antioxidant Effects

**Figure 5:**
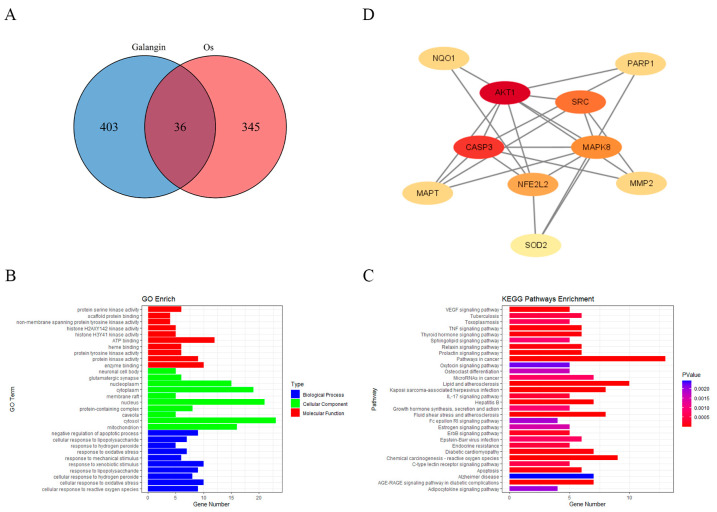
Prediction of the potential targets of GAL through which it exerts antioxidant effects. (**A**) The Venn diagram shows the number of genes that are common to both GAL target genes and oxidative stress-related genes. (**B**,**C**) Gene Ontology and Kyoto Encyclopedia of Genes and Genomes enrichment analyses of the intersecting genes. (**D**) The protein–protein interaction networks encompassing the 10 core targets.

On the basis of the above experimental results showing the ability of GAL to scavenge free radicals, we wanted to further explore which proteins GAL interacts with to exert its antioxidant effects. A total of 439 gene targets of GAL were predicted using the SwissTargetPrediction, PharmMapper, and SuperPred databases (Supplementary Data S1). A total of 381 genes related to oxidative stress were subsequently identified from the GeneCards and Gene Ontology databases (Supplementary Data S2). The Venn diagram constructed using the “VennDiagram” package (v1.7.3) in R (v4.4.0) (Figure 5A) revealed 36 intersecting genes between targets of GAL and oxidative stress-related genes. The GO and KEGG enrichment analyses of these intersecting genes were performed, and the results are presented as histograms (Figure 5B,C). GO enrichment analysis revealed that the enriched biological processes were related mainly to cellular responses to reactive oxygen species, oxidative stress, and hydrogen peroxide (Figure 5B). The cellular components in which the intersecting genes were enriched were clustered mainly in the cytoplasm, nucleus, and mitochondria (Figure 5B). The molecular functions in which the intersecting genes were enriched included ATP binding, protein serine kinase activity, and protein tyrosine kinase activity (Figure 5B). KEGG analysis revealed that the intersecting genes were associated mainly with cancer pathways, lipids and atherosclerosis, reactive oxygen species induced by chemical carcinogens, fluid shear stress and atherosclerosis, and the AGE-RAGE signaling pathway in diabetic complications (Figure 5C). We subsequently constructed PPI networks of these intersecting targets using the STRING website. The PPI network was analyzed using Cytoscape 3.9.1 to identify the core targets using the median degree, specificity, and betweenness values. Eventually, 10 genes were identified as core targets: *AKT1*, *CASP3*, *SRC*, *NFE2L2* (*Nrf2*), *MPK8*, *NQO1*, *SOD2*, *MAPT*, *MMP2*, and *PARP1* (Figure 5D). In addition, we also performed GO and KEGG enrichment analysis on the other 403 target genes of GAL and constructed a PPI network map to explore the possible role of GAL in porcine parthenogenetic embryos besides its antioxidant effect (Figure S3). The results showed that the enriched biological processes were mainly related to the activation of multiple growth factor receptor signaling pathways. The enriched cellular components were mainly in the cell membrane and cytoplasm, and the molecular functions were related to ATP binding, protein kinase and phosphatase activities, and various cytokine receptor activities. KEGG analysis showed that the non-antioxidant target genes of GAL were related to the regulation of cell cycles, such as metabolic pathways, MAPK signaling pathways, and FoxO signaling pathways. The PPI network interaction map showed that GAL mainly acted on energy regulatory genes such as *PRKACA*, *MAPK family*, *NT5M*, *AKT2*, etc.

### 3.5. GAL Exerts Antioxidant Effects Through the Nrf2/Keap1 Signaling Pathway

**Figure 6:**
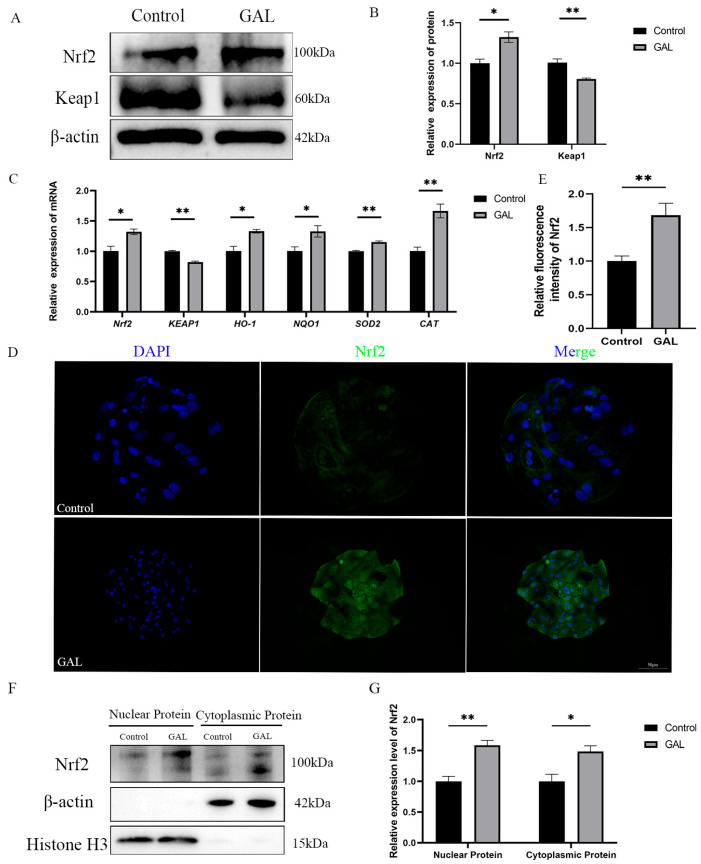
Effects of GAL supplementation on the Nrf2/Keap1 signaling pathway in porcine parthenogenetic embryos. (**A**) Western blot analysis of Nrf2 and Keap1 levels in the control and GAL-treated groups (*n* = 3). (**B**) Relative protein expression levels of Nrf2 and Keap1. (**C**) Measurement of the expression of genes related to the Nrf2/Keap1 signaling pathway in control and GAL-treated embryos by quantitative real-time reverse transcription polymerase chain reaction (*n* = 3). (**D**) Representative images of Nrf2 immunofluorescence staining in the control and GAL-treated groups. Scale bar = 50 µm. (**E**) Relative fluorescence intensity of Nrf2 (control: *n* = 24; GAL: *n* = 28). (**F**) Western blot analysis of Nrf2 expression levels in the nucleus and cytoplasm of cells in the control group and GAL-treated group (*n* = 3). (**G**) Relative protein expression levels of Nrf2 in the nucleus and cytoplasm. Significant differences are indicated by * (*p* < 0.05) and ** (*p* < 0.01).

Flavonoids have a wide range of antioxidant effects. Nrf2 is a key factor in regulating oxidative stress. Considering the experimental results described above, we hypothesized that GAL could interact with Nrf2 to promote the nuclear localization of Nrf2 to exert its antioxidant effects. To verify this role, we quantified the expression levels of *Nrf2* and its downstream genes. The results revealed that at the mRNA level, the expression of *Nrf2* was elevated in GAL-treated blastocysts (Figure 6C; *p* < 0.05); however, the expression of *Keap1* was reduced (Figure 6C; *p* < 0.01), and the expression of genes downstream of this signaling pathway (Figure 6C; *HO-1*, *p* < 0.05; *NQO1*, *p* < 0.05; *SOD2*, *p* < 0.01; *CAT*, *p* < 0.01) was also elevated. At the protein level, the expression level of Nrf2 in the GAL-treated group was greater than that in the control group (Figure 6A,B; *p* < 0.05), whereas the expression of Keap1 was reduced (Figure 6A,B; *p* < 0.01). The immunofluorescence results also revealed a significant increase in Nrf2 expression in GAL-treated blastocysts (Figure 6D,E; *p* < 0.01). After being released by Keap1 in the cytoplasm, Nrf2 must enter the nucleus to exert its function of promoting the expression of downstream antioxidant enzyme genes. To more intuitively demonstrate the activation of Nrf2 function in embryos after GAL treatment, we isolated the nuclear and cytoplasmic fractions of blastocysts and quantified the protein levels of Nrf2 in both fractions. The experimental results showed that after GAL treatment, the protein levels of Nrf2 in both the nuclear and cytoplasm of embryos significantly increased (Figure 6F,G; Nuclear Protein, *p* < 0.01; Cytoplasmic Protein, *p* < 0.05). These results indicate that the Nrf2/Keap1 signaling pathway was activated after GAL treatment.

### 3.6. GAL Enhances Protein Stability by Interacting with the Nrf2-Neh1 Domain

**Figure 7:**
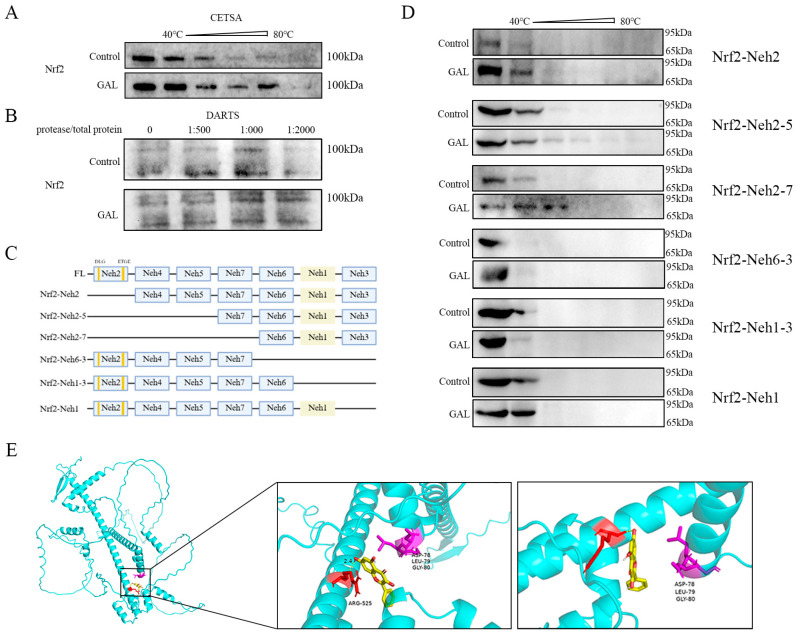
Identification of the structural domain through which GAL interacts with Nrf2. (**A**) Resulting of the CETSA showing the effect of GAL on the temperature resistance of embryos to Nrf2. (**B**) Result of the DARTS assay showing the effect of GAL on Nrf2 protease resistance in embryos. (**C**) Schematic representation of the Nrf2 structural domain and deletion mutants. (**D**) Schematic representation of the results of the CETSA showing temperature resistance in different mutants lacking different structural domains. (**E**) Molecular docking showing the site through which GAL interacts with Nrf2 and the location of the DLG motif. Yellow: galangin; red: Arg525 of the Neh1 domain; purple: “DLG” sequence of the Neh2 domain.

Small-molecule drugs can increase protein stability by forming complexes with target proteins. To investigate whether GAL directly binds to Nrf2 and regulates its function, multiple experiments were performed. First, the thermal stability of Nrf2 in porcine blastocysts was assessed using a CETSA (Figure 7A), and it was found that the GAL treatment significantly delayed the thermal denaturation of Nrf2 within the range of 40–60 °C. A DARTS assay further revealed that the degradation of Nrf2 by trypsin was reduced by GAL pretreatment (Figure 7B), indicating that the drug directly bound and maintained the structural integrity of Nrf2. To identify the binding region, six Nrf2 mutants lacking structural domains were constructed in this study (Supplementary Data S3 and Figure 7C), and in vitro CETSA experiments revealed that the ability of GAL to stabilize Nrf2 under hot conditions was completely abolished when the Neh1 domain of was missing, suggesting that the Neh1 domain of Nrf2 is the core binding region of GAL (Figure 7D). Molecular docking (AutoDock Vina) simulations revealed that GAL formed a hydrogen bonding network with Arg525 of the Neh1 domain (binding energy ΔG = −6.6 kcal/mol) (Figure 7E). These docking analysis results corroborate the experimental results described above and identify the precise site of action of GAL. In conclusion, GAL enhances the conformational stability of Nrf2 by directly binding to the Neh1 domain through hydrogen bonding.

### 3.7. The Antioxidant Effects of GAL Are Abolished After Nrf2 Inhibition 

**Figure 8:**
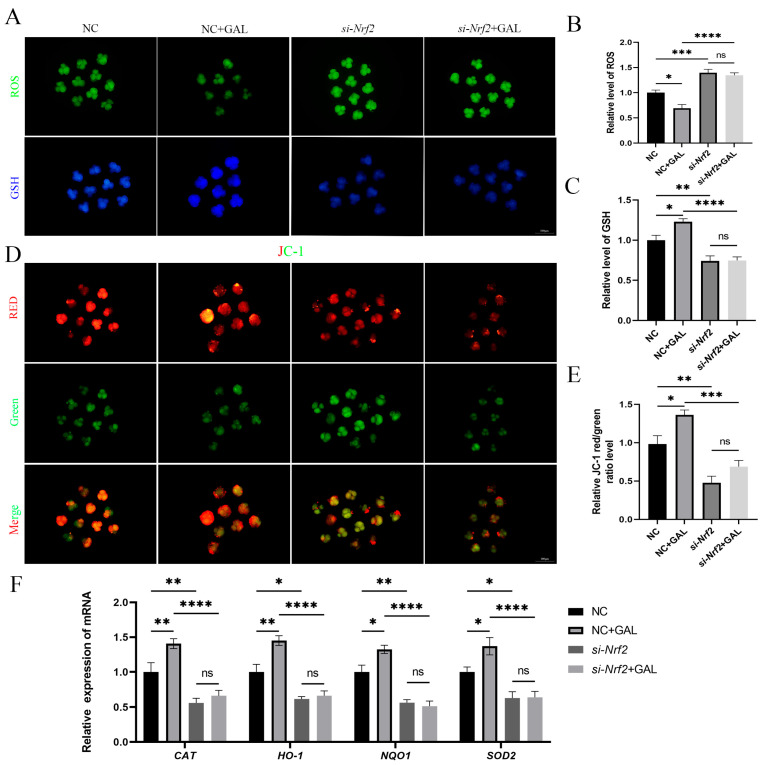
Effects of GAL supplementation after Nrf2 inhibition on antioxidant capacity and mitochondrial function in porcine parthenogenetic embryos. (**A**) Fluorescence images of intracellular ROS and GSH levels in control embryos, GAL-treated embryos, embryos with Nrf2 inhibition, and embryos with Nrf2 inhibition supplemented with GAL at the four-cell stage. Scale bar = 100 µm. (**B**) Relative ROS levels in the four groups (*n* = 50 per group). (**C**) Relative GSH levels in the four groups (*n* = 45 per group). (**D**) Fluorescence images of the mitochondrial membrane potential in the four groups at the four-cell stage. Scale bar = 100 µm. (**E**) Relative fluorescence intensity of a mitochondrial membrane potential marker in the four groups at the four-cell stage (*n* = 52 per group). (**F**) Measurement of the levels of antioxidant-related genes in the four groups by quantitative real-time reverse transcription–polymerase chain reaction. Significant differences are indicated by * (*p* < 0.05), ** (*p* < 0.01), *** (*p* < 0.001), and **** (*p* < 0.0001). No significant difference (*p* > 0.05) is indicated by “ns”.

To verify the intraembryonic interaction of GAL with Nrf2, *si-Nrf2* was microinjected into embryos after parthenogenetic activation, and GAL was subsequently applied during IVC to explore whether it exerts an antioxidant effect. The ability of GAL to scavenge ROS was abolished upon Nrf2 inhibition (Figure 8A,B; NC vs. *si-Nrf2*, *p* < 0.001; *si-Nrf2* vs. *si-Nrf2* + GAL, *p* > 0.05). In addition, GSH levels did not recover in the absence of Nrf2 (Figure 8A,C; NC vs. *si-Nrf2*, *p* < 0.01; *si-Nrf2* vs. *si-Nrf2* + GAL, *p* > 0.05). GAL failed to prevent the decrease in the mitochondrial membrane potential in the absence of Nrf2 (Figure 8D,E; NC vs. *si-Nrf2*, *p* < 0.01; *si-Nrf2* vs. *si-Nrf2* + GAL, *p* > 0.05). We subsequently examined the expression of relevant antioxidant enzymes in embryos and found that the expression of these enzymes decreased after Nrf2 inhibition and could not be increased even when GAL was applied (Figure 8F; *CAT*, *NQO1*; NC vs. *si-Nrf2*, *p* < 0.01; *si-Nrf2* vs. *si-Nrf2* + GAL, *p* > 0.05; *HO-1*, SOD2; NC vs. *si-Nrf2*, *p* < 0.05; *si-Nrf2* vs. *si-Nrf2* + GAL, *p* > 0.05). However, in the positive control group (NC + GAL), the antioxidant effect of GAL still existed and played a role in protecting mitochondria (Figure 8B,C,E,F). These experimental results suggest that GAL exerts its antioxidant effects through direct interaction with Nrf2.

## 4. Discussion

Physiological levels of ROS are necessary for normal growth, development, and metabolic activity in embryos [37,38]. The production of ROS by embryos is maintained at normal levels in vivo by a combination of exogenous antioxidants in the environment and antioxidant enzymes produced by the embryo [39]. However, during IVC, because the nonenzymatic antioxidants normally present in follicular and oviductal fluids are lacking in the culture system [40], ROS accumulate during embryo allotment and affect blastocyst formation and implantation [41,42]. A variety of exogenous factors, such as the oxygen concentration, metal ions, and light exposure [43,44,45], induce ROS production in embryos during IVC. Metal ions can form complexes with superoxide anion radicals to generate hydroxyl radicals or activate hydrogen peroxide to generate hydroxyl radicals, resulting in increased ROS levels [46]. While exposure to light is necessary during IVC of embryos, it also inevitably induces the production of ROS [47]. Therefore, antioxidant screening is an important step in the optimization of embryo culture systems. The addition of various classical antioxidants, such as melatonin, L-carnitine, and alpha-lipoic acid, to culture medium has been shown to reduce ROS levels in embryos [48,49,50]. In this study, we investigated the effect of GAL on the development of porcine parthenogenetic embryos. The experimental results revealed that GAL could increase the blastocyst rate and reduce the level of oxidative stress in porcine embryos, and network analysis and subsequent experiments demonstrated that this optimizing effect was achieved through the direct interaction of GAL with Nrf2.

Flavonoids have a wide range of biochemical properties, and their antioxidant effects are widely studied [46]. The antioxidant effects of flavonoids depend on numerous factors, such as their structural type, their degree of hydroxylation, and the position of hydroxyl substitution [51]. There are several mechanisms underlying their antioxidant effects. Flavonoids can inhibit the production of free radicals by chelating metal ions (iron, copper, etc.) [52]. Flavonoids have also been shown to inhibit enzymes involved in ROS generation [53] and promote antioxidant defense mechanisms [54] to exert their antioxidant effects. The antioxidant effects of GAL have been reported in various cellular and animal models [16,55,56]. In a mouse ototoxicity model, GAL was found to protect against drug-induced hearing damage by reducing mitochondrial ROS production [57]. In addition, another study showed that the antiaging effects of GAL on the skin may be related to its antioxidant effects [58]. Other studies have shown that GAL can alleviate diabetic retinopathy by reducing oxidative stress damage [59]. Although GAL exhibits positive antioxidant effects, there is still insufficient understanding of its pharmacokinetics and safety [60]. Studies have shown that flavonoids exhibit a biphasic dose–response phenomenon, displaying either antioxidant or pro-oxidant effects depending on drug concentration and the environment in which they act, particularly in the context of ROS scavenging, where they typically follow a U-shaped curve [61]. At low concentrations, flavonoids can scavenge free radicals to exert their antioxidant effects [46]; however, at high concentrations, they may increase ROS production and induce oxidative stress, leading to DNA damage [62]. Therefore, using the appropriate dosage of medication for the embryo is crucial to achieving the desired effect. Consistent with the previous findings, our experimental results indicate that GAL also exhibits an inverted U-shaped protective effect on porcine parthenogenetic embryos. At low concentrations (0.1 μM, 0.25 μM, 0.5 μM, 1 μM), as GAL concentration increases, its antioxidant effect becomes more pronounced, reaching its optimal effect at 1 μM. At this concentration, GAL significantly alleviates oxidative stress damage in porcine parthenogenetic embryos, with a notable decrease in ROS levels, an increase in GSH levels, and improvements in blastocyst formation rate, blastocyst cell count, and overall blastocyst quality. Beyond the optimal concentration (2 μM, 4 μM, 10 μM, 20 μM), GAL exhibits reduced protective effects on embryos and may even impair embryonic development. At this point, flavonoid compounds induce the production of highly reactive free radicals within the embryo, disrupting the electron transport chain and leading to damage to the embryonic membrane and DNA [63]. On the other hand, as GAL concentration increases, the metabolic burden on the embryo increases, impairing its normal developmental process and leading to embryonic developmental arrest. When GAL concentration rises to a certain level (40 μM, 80 μM), it exhibits drug toxicity, directly causing embryonic death. In clinical applications, the use of antioxidants in the culture medium to improve the quality of blastocysts is highly important for the subsequent implantation of embryos and for increasing pregnancy rates [64]. It has been shown that several flavonoids have high antioxidant activity, but there is a great deal of variation in their structures [65]; more research is still needed to demonstrate the key structures involved in the antioxidant effects of flavonoids.

Using network pharmacology to identify the targets of action of GAL and their overlap with antioxidant genes, we ultimately identified 10 core targets. Since Nrf2 is a major regulator of intracellular redox homeostasis and controls the synthesis of multiple downstream enzymes, we ultimately focused our research on Nrf2. Nrf2 can maintain glutathione homeostasis by directly regulating enzymes involved in glutathione synthesis. In addition, Nrf2 regulates a variety of ROS detoxification enzymes, such as glutathione peroxidase 2 (GPX2) and glutathione S-transferase [66,67,68]. Several experimental studies have shown that GAL can exert protective effects on cells or tissues through the Nrf2/Keap1 signaling pathway; for example, GAL enhances the activation and nuclear accumulation of Nrf2 and thus exerts a protective effect on the skin, and the antioxidant and antiaging effects of GAL are reversed after siRNA-mediated knockdown of Nrf2 [16]. Another study showed that GAL activates Nrf2 in human keratinocytes, leading to elevated expression of GSH synthase [69]. In addition, GAL inhibits oxidative stress through the Nrf2/HO-1 signaling pathway, thus ameliorating adriamycin (doxorubicin, DOX)-induced cardiotoxicity in mice [70]. Our experimental results revealed that applying GAL to porcine parthenogenetic embryos resulted in elevated expression of the Nrf2 protein in both the nucleus and cytoplasm, while decreased expression of the Keap1 protein and the mRNA levels of genes associated with the Nrf2-Keap1 pathway (*HO-1*, *NQO1*, *SOD2*, and *CAT*) were also elevated. HO-1 is encoded by the *HMOX* gene and is expressed in most organisms [71]. HO-1 reduces apoptosis by reducing ROS production [72,73]. In addition, HO-1 protects cells from damage by regulating mitochondrial function [74]. NQO1, a widely distributed FAD-dependent flavoprotein with multiple protective effects, utilizes NADPH or NADH as a hydrogen donor to reduce quinone compounds to hydroquinone with two electrons. The hydroquinone produced by this reaction is further metabolized as a sulfate coupler [75]. In addition, NQO1 directly scavenges superoxide, which is important for protecting some types of tissues and cells in which NQO1 is expressed at low levels [76]. SOD2 converts cellular superoxide anions to hydrogen peroxide [77], and CAT breaks down hydrogen peroxide into water and oxygen [78]. The increased mRNA expression levels of *HO-1*, *NQO1*, *SOD2*, and *CAT* in the present study suggest that treatment with GAL promotes the oxidative defense mechanisms of the embryo. Functional analysis of GAL’s non-antioxidant target genes showed that in addition to its antioxidant effect on the embryo, GAL can also regulate the embryo by regulating energy metabolism, cell cycle, signal transduction, and other functions during embryo development.

By constructing mutants lacking different structural domains, we identified that the structural domain through which GAL interacts with Nrf2 is the Neh1 domain. The presence of a basic leucine zipper structural domain (bZip) in the Neh1 domain allows for the formation of dimers with the sMAF protein and enhances the affinity of Nrf2 for the ARE, in addition to the ability of the Neh1 domain to participate in the regulation of nuclear translocation [79,80,81]. The binding of GAL to the Neh1 domain optimizes its binding to the sMAF protein and facilitates the entry of Nrf2 into the nucleus. It has been previously shown that GAL binds to Nrf2 with high affinity and dose-dependently inhibits Keap1 expression. In the present study, via molecular docking simulations, the hydroxyl group of GAL was found to bind to Arg525 in Nrf2. The spatial structure revealed that Arg525 in the Neh1 domain of and the DLG structure in the Neh2 region of Nrf2 are close to each other. Considering the experimental results of the present study, we hypothesized that after GAL binds to Arg525, the benzene ring structure of GAL blocks the binding of Keap1 to the DLG motifs, thus enabling the activation and accumulation of Nrf2. The activation of Nrf2 promotes antioxidant mechanisms in the embryo. The loss of the antioxidant effect of GAL after the inhibition of Nrf2 further validated the direct interaction between GAL and Nrf2. More studies are still needed to determine the specific amino acid residues involved in and the mode of action of the interaction between GAL and Nrf2, and how the combination of these two proteins prevents Keap1 from functioning.

As intracellular organelles, mitochondria synthesize ATP mainly through oxidative phosphorylation and play a role in providing energy during embryonic development [82,83]. Mitochondria are also important sources of ROS in the cell [55]. Under physiological conditions, free radicals produced by the mitochondrial respiratory chain and intracellular antioxidant defense mechanisms balance each other to ensure that mitochondria are not damaged [84]. When his balance is disrupted, the hydrogen atoms of unsaturated fatty acids on the mitochondrial membrane are used by ROS to form lipid free radicals, leading to the oxidation of lipids and the generation of harmful products [85,86]. These oxidized products further damage the mitochondrial membrane, impairing membrane fluidity and integrity and ultimately leading to mitochondrial dysfunction. Mitochondrial dysfunction manifests as changes in the membrane potential and a decrease in the number of mitochondria due to the excessive accumulation of ROS in the cell [83,87]. The results of the present study revealed that the mitochondrial number and membrane potential were significantly elevated by the addition of GAL during IVC of embryos. This finding is consistent with previous findings that mitochondrial dysfunction in the liver is alleviated and that the levels of mitochondrial enzymes and respiratory chain enzymes are normalized after the administration of GAL to hyperglycemic rats [55]. In cells exposed to H_2_O_2_/UVB and then treated with GAL, mitochondrial ROS production and the decrease in the mitochondrial membrane potential are alleviated [88]. The effect of GAL on mitochondria may be attributed to its uncoupling effect, through which it reduces the generation of superoxide radicals by the respiratory chain, thus preventing the excessive accumulation of ROS in the embryo [89].

In order to better evaluate the protective potential of GAL, it is important to compare its effects with those of other well-established antioxidants that have been used during IVC embryo, such as resveratrol, melatonin, and vitamin C. Previous studies have shown that these antioxidants exert beneficial effects through various mechanisms. For instance, resveratrol enhances SIRT1 expression and improves blastocyst development by reducing oxidative stress [90]; melatonin increases intracellular GSH levels, protects against DNA damage, and improves the inner cell/mass ratio [91]; while vitamin C reduces ROS and apoptosis, thereby enhancing overall embryo quality [92].

In comparison, GAL not only effectively reduces ROS and enhances antioxidant capacity but also activates the Nrf2 signaling pathway through interaction with its Neh1 domain. This multi-targeted mechanism may offer advantages over other antioxidants that primarily act through ROS scavenging. These findings suggest that GAL represents a promising candidate for improving the quality of in vitro embryo culture systems. Further research could explore the synergistic or additive effects of GAL in combination with other antioxidants to optimize embryonic development.

## 5. Conclusions

The addition of GAL during IVC of porcine parthenogenetic embryos can effectively reduce the level of oxidative stress in embryos, thereby improving the developmental quality of in vitro embryos. These effects are realized through the direct interaction of GAL with Nrf2.

## Figures and Tables

**Table 1:** The tested genes, primer sequences, PCR product sizes, and accession numbers for RT-qPCR experiments are provided in the following table.

Gene Name	Sequence	Amplicon Size (bp)	Accession Number
*Nrf2*	F: ATCCAGCGGATTGCTCGTAG R: TCAAATCCATGTCCTTGGCG	155	XM_013984303.2
*KEAP1*	F: ATGGCGGGGCCTCTGA R: CTCAGGGGCAGAAATTGGGT	114	NM_001114671.1
*HO-1*	F: GGCTGAGAATGCCGAGTTCA R: GTGGTACAAGGACGCCATCA	88	NM_001004027.1
*NQO1*	F: GTATAAAGTAGCCGGGCGCT R: AGTGCTTTTCTGACCGCCAT	162	NM_001159613.1
*SOD2*	F: AGGCGCTGAAAAAGGGTGAT R: AAGTCGCGTTTGATGGCTTC	163	NM_214127.2
*CAT*	F: GCTGAGTCCGAAGTCGTCTA R: GTCAGGATATCAGGTTTCTGCG	173	NM_214301.2
*β-actin*	F: TTCTAGGCGGACTTGCAGC R: GCTTCTCAGCAGACAGGAGG	128	XM_021086047.1

## Data Availability

Data available on request from the authors.

## Supplementary Materials

The following supporting information can be downloaded at: https://www.mdpi.com/article/10.3390/antiox14070822/s1, Supplementary Data S1: GAL Predictive Target Collection; Supplementary Data S2: Oxidative Stress Target Collection; Supplementary Data S3: Nrf2 Mutant Plasmid Sequencing Collection; Figure S1: The effect of different concentrations of GAL on embryo cleavage rate; Figure S2: The effect of different concentrations of GAL on blastocyst rate.; Figure S3: Analysis of GAL non-antioxidant target genes.
