# Supplementary material for: Galangin Regulates Oxidative Stress Levels in Porcine Embryos Through Interaction with the Neh1 Domain of Nrf2
Source: Antioxidants (Basel). 2025 Jul 4;14(7):822. doi: 10.3390/antiox14070822 (PMC12291769; doi:10.3390/antiox14070822)
Supplement: Supplementary file 1 [file antioxidants-14-00822-s001.zip › antioxidants-3697731-supplementary/Supplementary Figure.pdf]

Supplementary Figure:

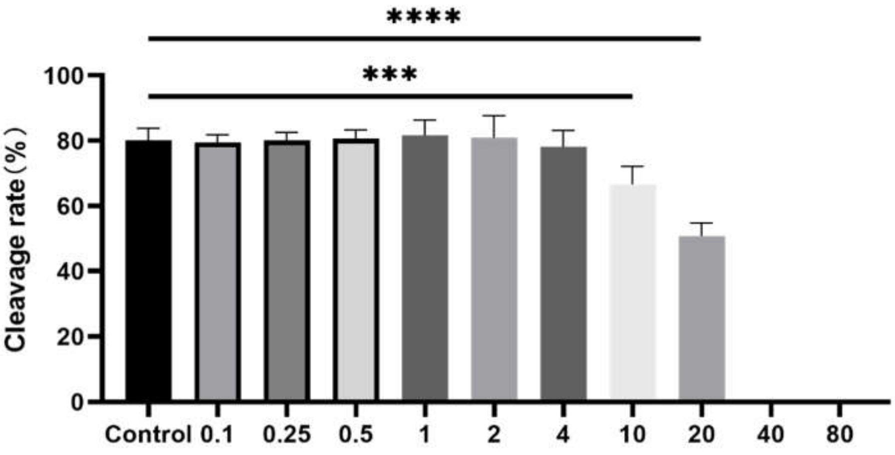

Figure. S1. The effect of different concentrations of GAL on embryo cleavage rate. Cleavage rate of porcine parthenogenetic embryos after 24 h (control: *n* = 262; 0.1 μM: *n* = 155; 0.25 μM: *n* = 160; 0.5 μM: *n* = 243; 1 μM: *n* = 265; 2 μM: *n* = 270; 4 μM: *n* = 266; 10 μM: *n* = 242; 20 μM: *n* = 182; 40 μM: *n* = 152; 80 μM: *n* = 155). Significant differences are indicated by *** ($P < 0.001$) and **** ($P < 0.0001$).

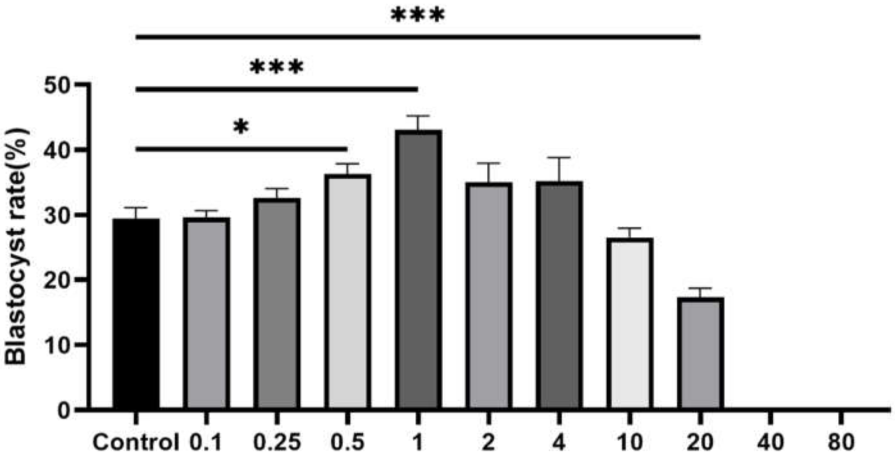

Figure. S2. The effect of different concentrations of GAL on blastocyst rate. Blastocyst formation rate of porcine parthenogenetic embryos on day 6. (control: *n* = 262; 0.1 μM: *n* = 155; 0.25 μM: *n* = 160; 0.5 μM: *n* = 243; 1 μM: *n* = 265; 2 μM: *n* = 270; 4 μM: *n* = 266; 10 μM: *n* = 242; 20 μM: *n* = 182; 40 μM: *n* = 152; 80 μM: *n* = 155). Significant differences are indicated by * ($P < 0.05$) and *** ($P < 0.001$).

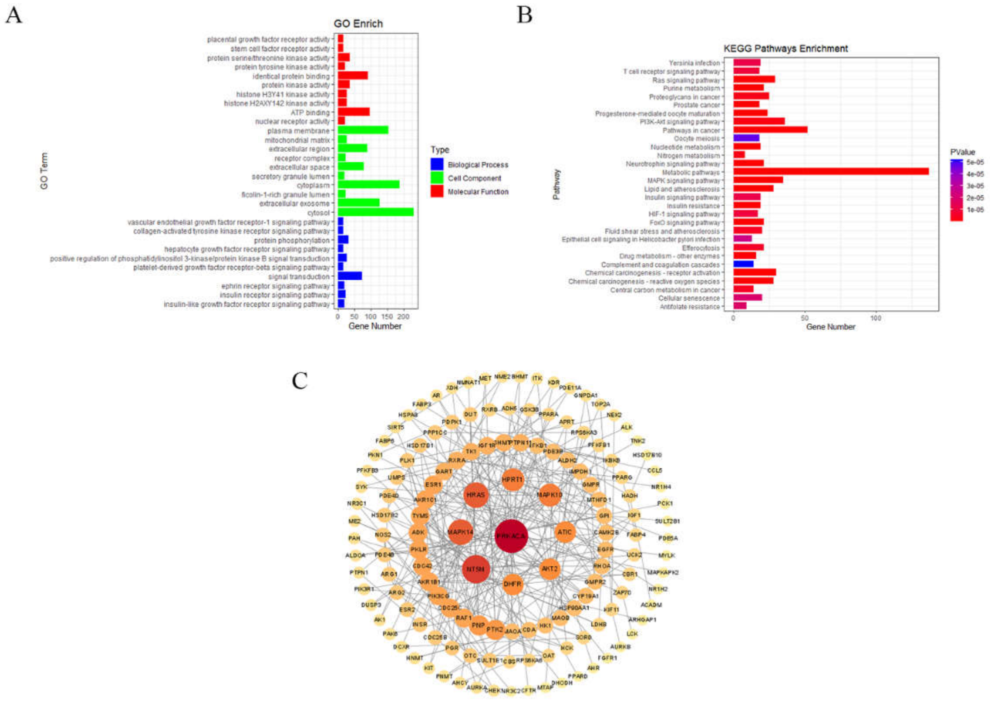

Figure. S3. Analysis of GAL non-antioxidant target genes. (A) and (B) Gene Ontology and Kyoto Encyclopedia of Genes and Genomes enrichment analyses of GAL non-antioxidant target genes. (C) The protein–protein interaction network encompassing the GAL non-antioxidant target genes.
